# Understanding Preferences for Lifestyle-Focused Visual Text Messages in Patients With Cardiovascular and Chronic Respiratory Disease: Discrete Choice Experiment

**DOI:** 10.2196/26224

**Published:** 2021-09-20

**Authors:** Michael Choi, Rebecca Raeside, Karice Hyun, Stephanie R Partridge, Aravinda Thiagalingam, Julie Redfern

**Affiliations:** 1 Westmead Applied Research Centre Faculty of Medicine and Health University of Sydney Sydney Australia; 2 Consumer Engagement and Codesign Research Hub Faculty of Medicine and Health University of Sydney Sydney Australia; 3 Department of Cardiology Concord Repatriation General Hospital Sydney Australia; 4 Prevention Research Collaboration Charles Perkins Centre University of Sydney Sydney Australia; 5 Department of Cardiology Westmead Hospital Sydney Australia; 6 George Institute for Global Health University of New South Wales Sydney Australia; 7 Research Education Network Western Sydney Local Health District Sydney Australia

**Keywords:** mHealth, cardiovascular disease, respiratory disease, visual communication, lifestyle change, consumer preferences, secondary prevention, rehabilitation, persuasive health technology

## Abstract

**Background:**

Supporting healthy lifestyle changes is a key aim of cardiovascular and pulmonary rehabilitation programs. SMS text messaging programs have demonstrated effectiveness in cardiovascular disease risk reduction, weight loss, increasing physical activity, and smoking cessation. The optimization of SMS text messaging programs may deliver greater population benefits as mobile phone use becomes ubiquitous. Visual messaging (ie, image-based messages) has the potential to communicate health messages via digital technology and result in enhanced engagement.

**Objective:**

This study aims to determine and understand patient preferences for lifestyle-focused visual text messages that support cardiovascular and pulmonary rehabilitation.

**Methods:**

A discrete choice experiment was conducted in a 4-stage iterative process to elicit patient preferences for visual message features. Attribute and level development yielded 3 attributes (purpose, image type, and web address), and 16 choice sets were subsequently constructed according to a full factorial design. Patients participating in cardiovascular and pulmonary rehabilitation were surveyed (on the web) for their preferences regarding the visual message choice sets. Respondents were asked to choose among 16 pairs of visual messages regarding key lifestyle behaviors, namely, physical activity and nutrition. The data were analyzed using a conditional logit model.

**Results:**

There was a total of 1728 observations from 54 unique respondents. Two factors that were associated with patient preference were gain-framed purpose compared with no purpose (odds ratio [OR] 1.93, 95% CI 1.40-2.65) and real images compared with cartoon images (OR 1.26, 95% CI 1.04-1.54). A loss-framed purpose was less preferred than no purpose (OR 0.55, 95% CI 0.42-0.74). Overall, patients preferred positive images that were colorful and engaged with text that supported the image and had a preference for images of real people rather than cartoons.

**Conclusions:**

A discrete choice experiment is a scientific method for eliciting patient preferences for a visual messaging intervention that is designed to support changes in lifestyle behaviors. SMS text messaging programs that use visual aids may result in greater patient satisfaction by using a gain frame, using real images, and avoiding a loss frame. Further research is needed to explore the feasibility of implementation and the health and behavioral outcomes associated with such visual messaging programs.

## Introduction

### Background

Noncommunicable diseases (NCDs) are the leading cause of disease burden worldwide [1]. Two major NCDs—cardiovascular disease (CVD) and chronic respiratory disease—have negative impacts on quality of life [1], health care costs [2], and worker productivity [3,4]. NCDs are generally defined by their persistent effects on health, which require long-term management [5]. NCD disease burden is largely manageable by addressing the following four key modifiable risk factors: tobacco use, alcohol misuse, physical inactivity, and diet [5]. Chronic disease management programs, also known as rehabilitation, offer group exercise and education sessions with the aim of improving key modifiable risk factors [6,7]. The benefits of both cardiac and pulmonary rehabilitation are well evidenced, which include reduced mortality, improved quality of life, and reduced hospital admissions [7,8]. However, uptake and attendance rates are poor, estimated to be between 10% and 30% globally [9,10] because of logistical barriers of time and distance [11,12].

The rapid advancement of mobile technology presents an opportunity to increase participation in existing rehabilitation programs and support self-management. There are >4.7 billion mobile phone users worldwide [13], and SMS text messaging has become an inexpensive and simple way for people to communicate. Strong evidence supports the effectiveness of SMS text messaging interventions to promote smoking cessation [14], weight loss [15], and increased physical activity [16]. A randomized controlled trial is currently underway to evaluate the effectiveness of a text message support program for patients with CVD and chronic respiratory disease [17]. This is based on a previous randomized controlled trial, the Text Me study, which showed that a lifestyle-focused SMS text messaging intervention was effective in improving cardiovascular risk factors in patients with coronary heart disease [18]. Given that visual aids have been found to enhance the engagement and understanding of health messages [19], it is possible that a visual messaging program could demonstrate similar, if not better, outcomes to previous SMS text messaging interventions for different populations; however, this is a relatively unexplored area. Researchers have explored the application of visual aids in technologies aimed at healthy behavior change, such as websites [20,21] and mobile apps [22-24]. However, there is a paucity of research investigating how visual aids may look like in a mobile messaging format. To the best of our knowledge, only one other study [25] has examined whether visual-based mobile phone messages could be used to support healthy lifestyle behavior change. However, this was tested in a cohort of young, healthy university students with no chronic disease, and the messages were stylistically simple. Therefore, we explored the feasibility of an infographic-style visual message containing both images and text. As an infographic-style visual message is made up of many characteristics (eg, color, type of image, and intention), we also investigated patient preferences with regard to the differing characteristics of this specific format. This project, which explored the use of visual aids in mobile messaging, represents a novel expansion in the growing field of persuasive health technology, which aims to use technology to support positive behavior change [26].

Understanding patient preferences regarding various aspects of SMS text messaging interventions is important for improving them. Qualitative methods such as focus groups are commonly used to gather patients’ perspectives, as used by the authors of the Text Me trial to investigate why participants found the intervention useful [27]. These methods are robust; however, when trying to understand what patients like and dislike about certain features of infographic-style visual messaging (ie, whether they prefer this type of image over another), alternative methods may be better suited. For example, one SMS text messaging intervention used a quantitative method called a discrete choice experiment (DCE) to elicit patient preferences for various aspects, such as the frequency of messaging [28]. DCEs are a quantitative survey method and have been used to measure patient preferences for health care services and products [29]. DCEs define health care products and services by their attributes (ie, characteristics), where each attribute has various levels. For example, one attribute of a health care product could be price, and its levels could be US $1, US $2, and US $5. DCEs then present respondents with a choice set containing 2 or more alternatives to choose from, where each alternative is described by a different attribute-level combination. When a respondent chooses an option, their preferences for certain attributes can be estimated, and the relative weighting of attributes can be calculated [30]. Although this is a robust preference elicitation method, no DCEs to date have been used to explore the preferences for visual messaging in a population of patients with CVD and chronic respiratory disease.

### Objectives

Therefore, the overall aim of this study is to understand the preferences of patients with CVD and chronic respiratory disease for lifestyle-focused visual-based messages.

The first objective is to determine which visual messaging attribute levels were preferred by patients with CVD and chronic respiratory disease using a DCE. The second objective is to explore the reasons why patients preferred or not preferred certain visual messages using a single open-ended question.

## Methods

### Study Overview

**Figure 1:**
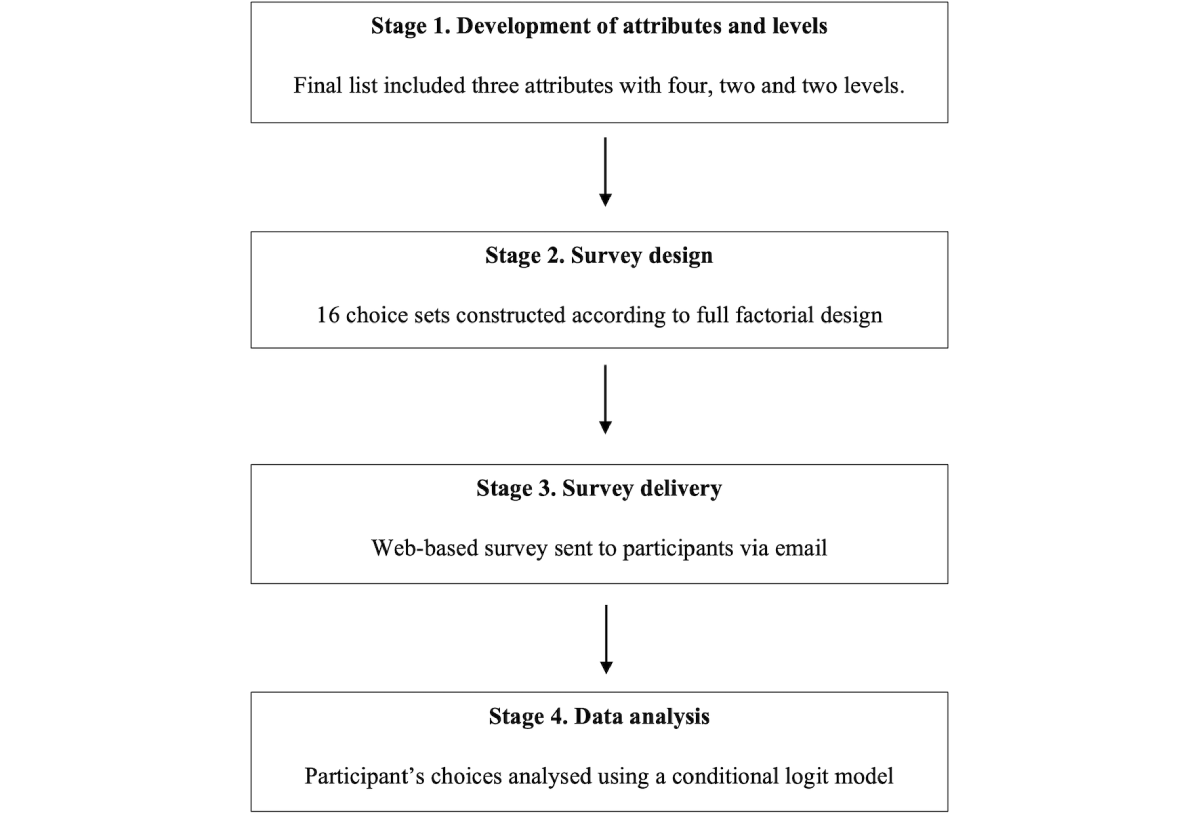
The four stages of the discrete choice experiment.

This study was conducted as part of the process evaluation component of an integrated text messaging (ITM) study [17]. Ethics approval was obtained from the Ethics Review Committee of the Sydney Local Health District (HREC/16/RPAH/362), Sydney, Australia. To address the first objective of determining which visual messaging attribute levels influenced participants’ choices, a DCE was conducted. This DCE followed the ISPOR (International Society for Pharmacoeconomics and Outcomes Research) guidelines on conjoint analysis methods [31]. The following stages were conducted and are described in the following sections: (1) the development of attributes and levels, (2) survey design, (3) survey delivery, and (4) data analysis (Figure 1). For addressing the second objective of exploring the reasons why certain visual messages were preferred or not preferred over others, a single open-ended question was asked after each choice set. This is described in the final section of the *Methods* section.

### Stage 1: Development of Attributes and Levels

The development of attributes and attribute levels was carried out in two steps. First, a list of potential attributes was generated by examining the literature and discussing with experts from SMS text messaging interventions (AT, JR, and RR) [32,33]. Eight attributes were identified on the basis that patients might find them preferable: education (simple steps on how to perform a behavior) [28], framing (explaining the positive or negative outcomes of performing a behavior) [34-36], call to action (a command to perform a behavior) [37], testimonial (stories of similar people performing certain behaviors), color, mood (the overall emotional feeling), URL (a link to a web address), and a credibility indicator (acknowledgment of information as coming from a credible source) [28]. After the attributes were identified, they were reviewed independently by the research team (AT, JR, and RR), and their suggestions were then incorporated into the revised list of attributes and levels. Following DCE guidelines [29], which recommend no more than 6 attributes to minimize survey burden, we combined similar defined attributes. For example, the *URL* and *credibility* attributes were combined into a new attribute, *URL to a credible source*, because the internet site used was of high quality. Where attributes could not be combined together, they were redefined as attribute levels. For example, the attributes *color*, *mood*, and *framing* were redefined as levels *gain-frame* and *loss-frame* underneath a new attribute called *purpose*. A visual message with the *gain-frame* attribute level was specified by bright, saturated colors to create a positive mood and contained text explaining the benefits of performing the health behavior. On the other hand, a visual message with the *loss-frame* attribute level was specified by dark, unsaturated colors to create a negative mood and contained text explaining the losses of not performing the health behavior. The final list of attributes and levels derived from this iterative process is outlined (Textbox 1).

**Included attributes and levels of this discrete choice experiment.**

**Message purpose**

- None (no additional information)
- Educational (simple instructions for performing health behaviors)
- Gain-frame (benefits of health action are explained)
- Loss-frame (losses of not performing health action are explained)

**Image type**

- Not real-life images (eg, cartoons, icons, and stick figures)
- Real-life images (eg, real-life people, objects, and places)

**URL link**

- Without (no web address provided)
- With (web address provided)

### Stage 2: Survey Design

**Figure 2:**
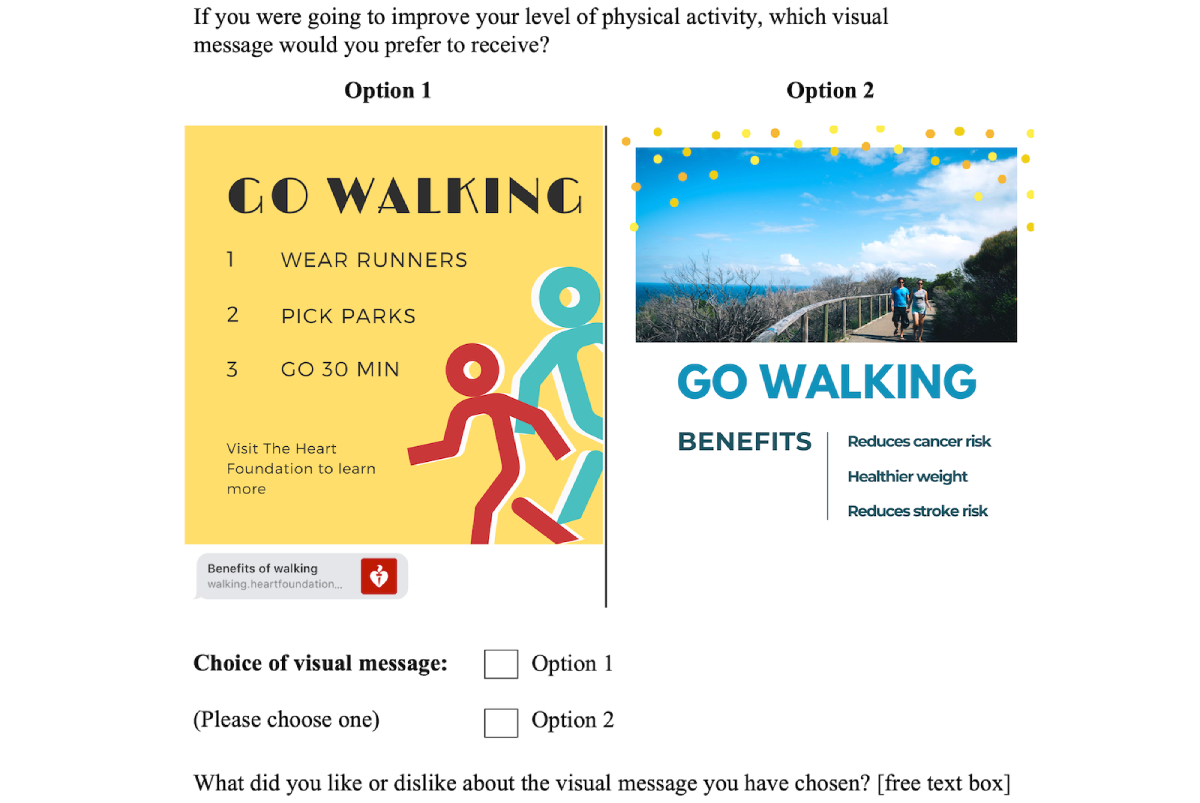
Example of a choice set from the discrete choice experiment survey.

**Figure 3:**
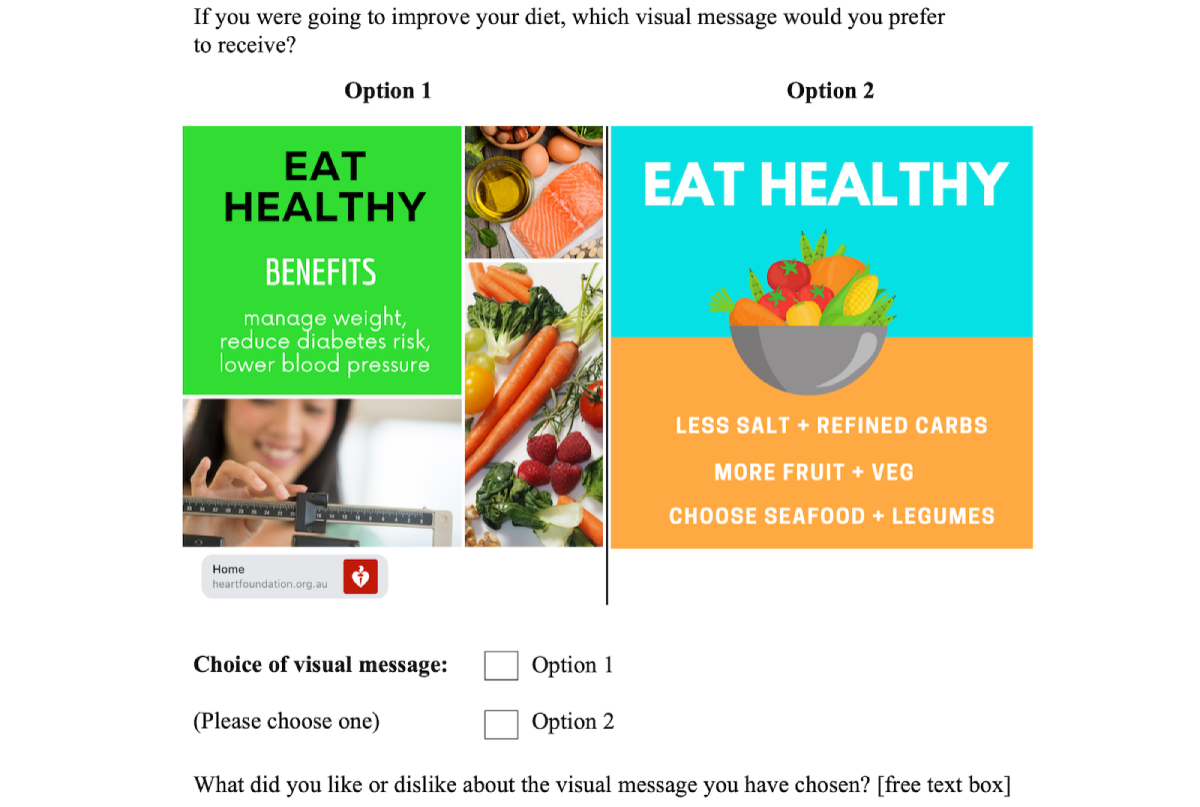
Example of a "go walking" choice set from the discrete choice experiment survey.

Given the small number of attributes and levels (3 attributes at 4, 2, and 2 levels), we decided to use a full factorial design consisting of 16 choice sets. However, no orthogonal array was available to fit this design, and so, the *idefix* package (Rstudio Inc v3.6.0) [38] was used to generate an appropriate survey design that randomized and balanced the various attribute-level profiles. The result was a DCE design with a DB-error (Bayesian D-error) efficiency of 0.4%. Once the survey design was specified, visual messages were crafted according to the required attribute-level combinations of each choice set. The content of the visual messages was sourced from the original SMS text messages developed for the ITM study (developed based on the published process) [17,39] along with stakeholder websites. The ITM SMS text messages themselves were developed based on the behavior change taxonomy of Michie et al [40]. Therefore, our visual messages were directly adapted from these original ITM SMS text messages. The first 8 choice sets centered around the message *go walking*, and the latter 8 centered around *eat healthy*. Visual messages were constructed according to the level specified for each attribute. For example, for the attribute levels *educational*, *nonreal*, and *with URL*, the actions required to follow the proposed health behavior would be explained, a cartoon image would be inserted, and a URL link would be attached (Figures 2 and 3). Similarly, for the attribute levels *gain-frame*, *real image,* and *without URL*, a real-life photograph would be inserted, and bright saturated colors would be used to emphasize the benefits of taking that certain health action (Figures 2 and 3). Different variations in font, sizing, and layout were considered for each visual message. After an iterative design process for refinement, 32 visual messages were selected. Each choice set was preceded by the question stem “If you were going to improve your diet or your level of physical activity, which visual message would you prefer to receive?”

### Stage 3: Survey Delivery

Before the delivery of the web-based survey to participants, it was pilot tested among the research team. The aim was to explore the acceptability of the DCE survey, the time taken to complete it, and the ease of understanding. Once refinements were made, a link to the web-based survey was sent to 141 participants via their nominated email address. These participants had already been recruited as part of the ITM study [17]. Eligible respondents consisted of adults with a medical history of CVD (including coronary heart disease, cardiomyopathy, peripheral arterial disease, and stroke) or chronic respiratory disease (chronic obstructive pulmonary disease, chronic bronchitis, emphysema, chronic asthma, and bronchiectasis). These participants were recruited from 6 rehabilitation clinics across Sydney, Australia. Eligible respondents were identified by clinical rehabilitation staff and invited to participate in the ITM study. Participants provided informed consent to participate in message feedback and improvement. Of the 141 participants, 54 (38.3%) people responded to the survey. Out of the 54 respondents, 6 (11%) respondents did not fully complete all questions.

### Stage 4: Data Analysis

#### Overview

Data analysis was informed by the ISPOR guidelines on appropriate statistical methods for DCEs [41]. Analyses were performed using SAS v9.4 (SAS Institute). Patient characteristics were summarized as mean and SD for continuous variables and number and percentage for categorical variables. To analyze the DCE, a conditional logit model, also known as McFadden’s choice model [42], was used, and odds ratios (ORs) and 95% CIs were estimated. The conditional logit model is based on the random utility theory, which states that choices are made because they bring utility to the individual [43]. It was assumed that the patients would choose the visual message (1 or 0) that maximizes their utility depending on the attributes. This was formulated as follows:

*U (1, 0)* = *β_1_ educational* + *β_2_ gain-frame* + *β_3_ loss-frame* + *β_4_ real image* + *β_5_ URL* + 𝜀 **(1)**

#### Single Open-ended Question

For addressing the second objective, participants were asked an open-ended question to explore the reasons why certain visual messages were chosen or not chosen over others. After each choice set, participants were asked, “What did you like or dislike about the visual message you have chosen?” Participants typed their responses in a free text box (Figures 2 and 3). An inductive thematic analysis was conducted to analyze these responses [44]. First, the research team familiarized themselves with the data by reading through the responses. Second, a set of initial codes was generated, and each response was tagged with one or more of these codes. As this was an iterative method, new codes were added as new elements came up in the data. Next, the codes were grouped under themes, which were defined as patterns that describe something significant or interesting about the data. These themes were then reviewed, and definitions were developed. Overlapping themes were separated, and subthemes were developed. The interactions between themes were also examined.

## Results

### Overview

We received 54 surveys that resulted in a total of 1728 observations.

### Respondent Characteristics

Most of the study sample (mean age 69.7 years, SD 8.7) was male (34/54, 62%), was of White ethnicity (43/54, 84%), and was diagnosed with CVD (32/54, 59%; Table 1). The median level of education in the cohort was diploma certification.

**Table 1:** Baseline demographic and clinical characteristics (N=54).

Demographic			Values
Age (years), mean (SD)			69.7 (8.7)
**Gender, n (%)**			
	Men	34 (62)	
	Women	20 (37)	
**Ethnicity^a,b^, n (%)**			
	White	43 (84)	
	Aboriginal or Torres Strait Islander, Chinese, Japanese, Malay, South Asian^c^, Arab or Persian, Black African, Sub-Saharan African, or Native American Indian	6 (12)	
	Other	2 (4)	
Education^a,d^, median (IQR)			5.0 (4.0-7.0)
**Clinical disease type, n (%)**			
	Diagnosed with CVD^e^	32 (59)	
	Diagnosed with chronic respiratory disease	22 (41)	

^a^As baseline data were collected from previous medical records, we collected all available data at the time of extraction.

^b^Ethnicity data were only available for 51 participants.

^c^Bangladesh, India, Nepal, Pakistan, or Sri Lanka.

^d^Education data were only available for 51 participants.

^e^CVD: cardiovascular disease.

### Visual Messaging Attribute Levels That Influenced Preferences

**Figure 4:**
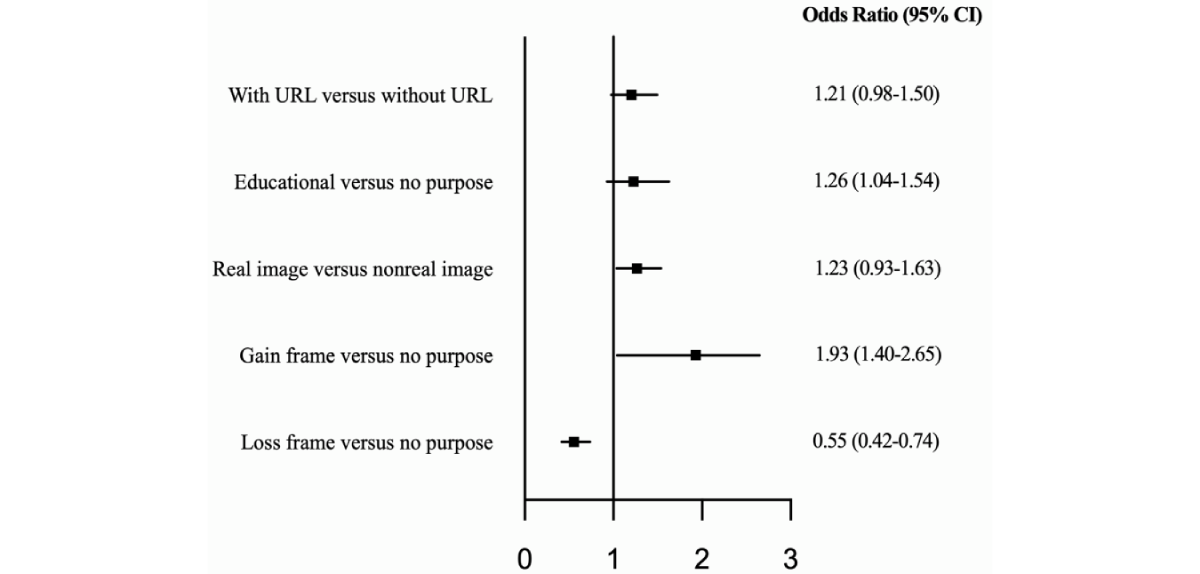
Attribute levels of visual messages, which influenced participant choice.

As per the first objective, a DCE was used to determine which visual messaging attribute levels influenced preferences. According to the conditional logit model, the following three attribute levels significantly influenced participant preferences: a gain frame, a loss frame, and real images. Visual messages with a gain-framed purpose were approximately 93% more likely to be chosen over messages with no specific purpose (OR 1.93, 95% CI 1.40-2.65; Figure 4). Conversely, visual messages with a loss-framed purpose were approximately 45% less likely to be chosen than messages compared with no purpose (OR 0.55, 95% CI 0.42-0.74). Visual messages with a real image were 26% more likely to be chosen than messages with a nonreal image (OR 1.26, 95% CI 1.04-1.54).

### Reasons for Choosing Certain Visual Messages

**Figure 5:**
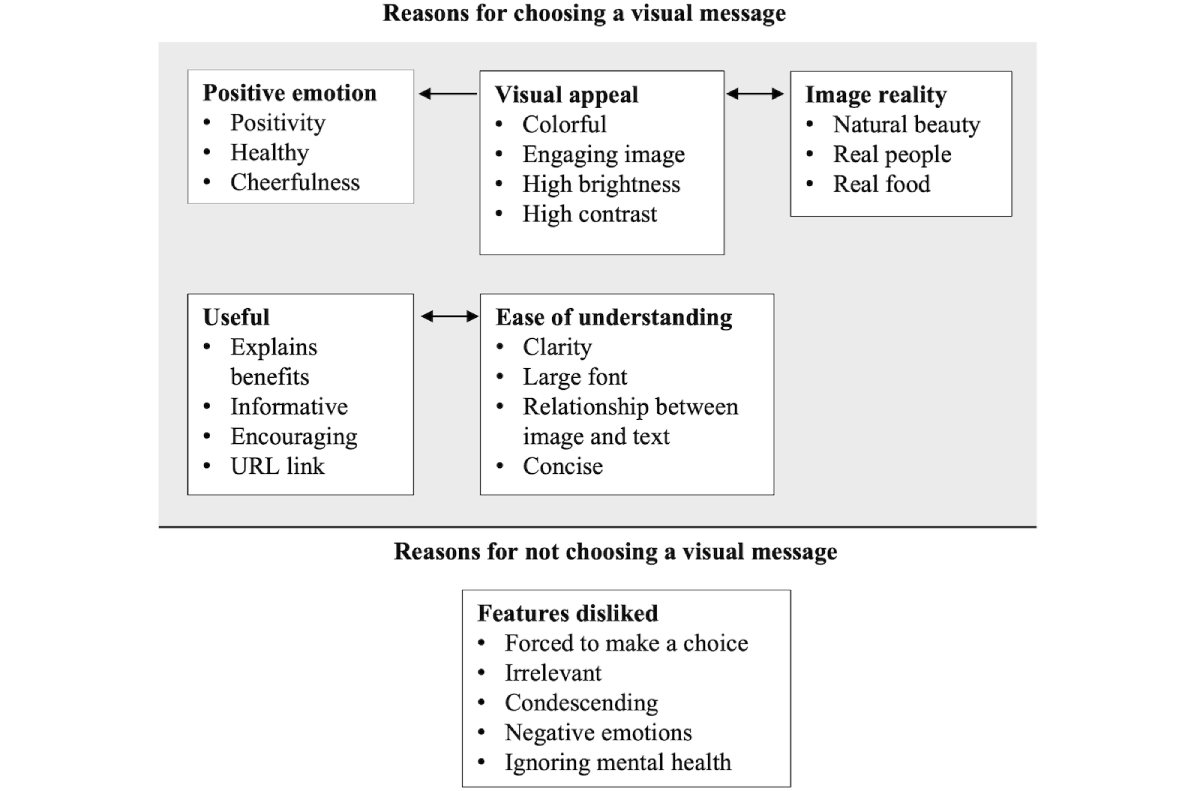
Illustrative quotations of what participants liked and disliked about the visual messages.

As per the second objective, an open-ended question was asked after each choice set to explore the reasons why certain visual messages were preferred over others. The inductive thematic analysis resulted in the following six themes and subthemes: positive emotions (positivity, healthy, and cheerfulness), visual appeal (colorful, engaging image, high brightness, and high contrast), ease of understanding (clarity, large font, relationship between image and message, and concise), usefulness (benefits explained, informative, encouraging, and URL link), image reality (natural beauty, real people, and real food), and disliked features (forced to make a choice, irrelevant, condescending, negative emotions, and ignoring mental health). Illustrative quotations are provided in Table 2, and the conceptual patterns and relationships among the themes are shown in Figure 5.

**Table 2:** Illustrative quotations of what participants liked and disliked about the visual messages.

Theme			Quotations		Participant characteristics (gender, age, and disease type)
**Positive emotions**					
	Positivity	“Stands out with a more positive vibe.”		Male, 56 years, COPD^a^	
	Cheerful	“Much cheerier–colour probably.”		Female, 66 years, CVD^b^	
**Visual appeal**					
	Colorful	“Bright colours encourage people to look, read and absorb idea.”		Male, 53 years, CVD	
	Engaging image	“Photo of good-looking food makes me want to eat better.”		Female, 57 years, COPD	
**Ease of understanding**					
	Simplicity	“Clear. Easy to read.”		Male, 29 years, CVD	
	Relationship between image and message	“Photos supported text clearly and effectively. Good clear message.”		Male, 48 years, CVD	
**Usefulness**					
	Benefits explained	“It explains what the things [benefits] that you can get from walking.”		Male, 43 years, COPD	
	URL link	“Links to Heart Foundation [website].”		Male, 36 years, CVD	
**Image reality**					
	Nature images	“The real outdoor picture instantly draws you to a feeling of health, fresh air and sunshine.”		Female, 42 years, COPD	
	Real people	“I’d rather see photos of people than cartoonish drawings. The other option is very unattractive!”		Female, 66 years, CVD	
**Features disliked**					
	Forced to make a choice	“Neither is that good. Need to give reason rather than lecture or order exercise.”		Male, 56 years, CVD	
	Condescending or offensive tone	“Both are terrible assuming people are idiots.”		Female, 57 years, CVD	
	Negative emotions	“Yet again try to stay away from negative images.”		Male, 40 years, CVD	

^a^COPD: chronic obstructive pulmonary disease.

^b^CVD: cardiovascular disease.

## Discussion

### Principal Findings

To our knowledge, this is the first study to examine the preferences of patients with CVD and chronic respiratory disease for varying characteristics of lifestyle-focused visual-based messages to be sent digitally. As per the first objective, this study used a DCE to determine whether 3 visual messaging attribute levels significantly influenced participant preferences: gain frame, loss frame, and real images. As per the second objective, this study analyzed the responses to a single open-ended question and discovered the reasons for choosing or not choosing certain visual messages. The reasons for choosing a visual message included positive emotion, visual appeal, image reality, ease of understanding, and usefulness. The reasons for not choosing a visual message included irrelevant information, condescending tone, and negative emotions. These findings have important design implications for infographic-style health messages, suggesting that gain-framed messages with lifelike photos are likely to be well received by patients with CVD and chronic respiratory disease.

### Comparison With Prior Work

This study found that framing had a strong influence on participants’ preferences; gain-framed messages were liked, and loss-framed messages were disliked. This proclivity toward gain framing and aversion to loss framing is a well-known phenomenon reported in the health communication literature. It is unclear why framing effects occur. Therefore, understanding how framing can effect preferences may inform future implementation of visual messaging programs. It has been suggested that the framing effect depends on the type of behavior being communicated. For example, Rothman and Salovey [45] discovered that gain-framed messages are more effective than loss-framed messages in promoting disease-preventing behaviors. In support of this idea, a meta-analytic review of 94 studies demonstrated that gain-framed messages were slightly more effective than loss-framed messages in promoting disease-preventing behaviors [46]. Rothman and Salovey [45] also discovered that loss-framed messages were more effective than gain-framed messages in promoting disease-detecting behaviors. They proposed that this occurs because of prospect theory [47], which states that people will be risk averse when considering actions with guaranteed benefits and risk seeking when considering actions with potential losses. In this study, two disease-preventing behaviors (walking and healthy eating) were communicated to participants, and it was found that gain-framed messages were preferred. In line with the work of Rothman and Salovey [45], we suggest that the observed framing effect is because of the fact that the behaviors being promoted are disease preventing and that if they were disease-detecting behaviors, then loss-framed messages would be preferred over gain-framed messages. Therefore, when incorporating framing appeals into health messages, it is important to consider what type of behavior is being advocated so that the message is well received by participants.

Another line of research has proposed that framing effects may depend on the individual’s disposition. For example, Covey [48] suggested that framed messages that are congruent with an individual’s motivation are likely to be preferred, that is, gain-framed messages are more persuasive when the recipient is reward oriented, and loss-framed messages are more persuasive when the recipient is threat aversive. It is unknown what the motivational status of this population is, although some evidence suggests that the patients undergoing cardiac rehabilitation are likely to be threat aversive as they have undergone a major coronary event and would want to avoid it again in the future [19]. In that case, the findings of this study contradict the findings of Covey [48]; however, this is difficult to tease out, as we do not know whether this population is threat aversive or reward oriented. Therefore, it is unknown whether the framing effects in this study are mediated by the disposition of the participants. Future research is needed to test whether the motivational status of people with chronic diseases might mediate framing effects.

In discussing framing effects, one study found little impact because of message framing. Niu et al [25] examined the impact of framed SMS text messages on encouraging healthy eating practices. They discovered that participants’ cognitive responses did not differ between gain-framed and loss-framed messages, that is, there was no observable difference because of framing. However, there are at least 3 factors that may explain the discrepancy in findings between this study and the study by Niu et al [25]. First, the studies had different aims; this study sought to understand one’s choices, and the other examined one’s cognitive processes. Second, different methodologies were used; this study used a DCE, and the other study used a questionnaire. Finally, the samples were different demographically and clinically; this study examined an older cohort with chronic disease, and the other study examined a group of young, healthy undergraduates. These three factors may explain why this paper discovered a strong response to framing and why Niu et al [25] saw virtually no response to framing.

Although framing was an influential design attribute, there were other factors that may have accounted for the large difference in preferences for gain-framed and loss-framed messages. For example, certain colors may have attracted some respondents to that particular visual message. In this DCE, bright saturated colors were used when designing gain-framed messages. In contrast, dark colors were used when designing loss-framed messages. The purpose of the color was to accentuate the respective gains and losses that the supposed action would bring. Color, hue, and saturation preferences have been demonstrated [19,49], which may have influenced the participants’ choices. The findings of the thematic analysis support the idea that messages with positive colors are preferred. Therefore, the strong preference for gain-framed messages was likely influenced by the use of positive colors.

Our results highlight that adapting messages into a digital visual format requires the consideration of several design features. For example, the use of images is a key design feature. As the digital age advances, capturing and communicating photographs will become easier and can be used in mobile health (mHealth) interventions. Although it is understood that visual media can impact the processes leading to behavior change [19,25], no studies to date have examined whether the type of image might have a different impact. This study found preferences for real images over nonreal ones in a population of cardiac and pulmonary rehabilitation patients. It is known that photos of people are powerful, persuasive tools as viewers can establish a relationship with the subject [50,51]. They elicit immediate emotional reactions that can affect their behavior. The effects of visual images on behavior change have also been reported when there are demographic similarities between the subject in the photograph and the viewer [52]. It is unknown whether this effect also applies to patients with chronic diseases. Future studies can examine whether visual image tailoring may influence the reception of visual messages.

Text is another important element of visual messages. In this study, we paired brief textual information with images. In general, participants preferred these messages to messages with no text as the text explained the visual message of the photo in a manner that was simple and easy to understand. This finding adds to the current understanding of effective infographic-style health messages. In fact, simplicity as a design principle has been identified as an important factor influencing user acceptability of mHealth technologies in patients with CVD [53]. However, messages that were too simplistic and contained no textual information were generally disliked. Instead, what patients seemed to prefer was a textual message that gave meaning to the visual aid in a clear and concise way. Factors such as font sizing, colors, layout, and positioning all contribute to how the text in a visual message is perceived and should be carefully considered to maximize clarity.

An interesting feature (further research to evaluate) used in this study’s visual messages was a URL link. The purpose of this was to act as a means for people to pursue more information. The presence of the URL link also serves as a credibility indicator. The addition of a URL link is a benefit of SMS text messaging as a communication modality. Not only can respondents see the website address, but those with internet capabilities are also able to follow the link immediately and view the website’s information. The findings from this study were aligned with those of past studies examining the role of visual aids in supporting chronic disease education. Visual aids have been used across a variety of formats, including print materials, websites, and mobile apps, to support the education of patients with CVD and chronic respiratory disease [20,22,24,54-57]. However, it is unknown whether visual-based messages can be used. The findings from this study suggest that there is great potential for visual messages that combine real images with a simple lifestyle-focused message to be well received by patients undergoing chronic disease rehabilitation.

The strength of this study lies in its DCE methodology. DCEs have many advantages over traditional surveys in eliciting preferences. First, DCE is an accepted survey method. The benefit is that best practice guidelines were able to be followed both in experimental design and also in statistical analysis [31,41]. Although the DCE originated in economics [30] and has been widely used to determine preferences for health services and products [29], this is the first instance of a DCE being used to explore the preferences for visual messaging. This only highlights the versatility of a DCE and its future potential to be used more widely in examining patient preferences in the field of mHealth interventions.

Another key advantage of DCE is its quantitative analysis. Rather than using a traditional questionnaire that yields qualitative results, DCE can measure preferences [29]. Of course, learning about patient preferences is enhanced by both qualitative and quantitative methods. In fact, qualitative methods such as focus groups are highly recommended when developing an initial idea of attributes, which the target population may care about [33]. The full power of the DCE is realized when the attributes are numeric. This allows for powerful analyses, where choice can be graphed as a dependent variable of attribute variables. However, in this study, the attributes were categorical variables (message purpose, image type, and URL link); thus, this type of analysis was not possible. Nevertheless, quantitative results were obtained in the form of an OR, allowing the relative size of the attributes to be ascertained.

DCE is also a robust method, as it indirectly measures preferences. Participants were asked to choose the message they preferred but not directly told of the different attributes that comprised each message. This reduced participants’ bias, as they have to consider which message they actually prefer by comparing the two. Alternative preference elicitation methods could have also been used. For example, multiprofile best-worst scaling is a method in which the participant is asked to rank several choices from best to worst [58]. A variant of this method is single-profile best-worst scaling, in which a participant is presented with a single option and asked to state the best and worst aspects. Therefore, the DCE is a robust quantitative method that holds great potential for future studies on patient preferences for mHealth interventions.

### Limitations

One of the limitations of this study was the lack of a no-choice alternative. Participants were offered to choose between two visual messages with no choice to opt out. This is an important limitation as thematic analysis of the free text responses revealed that some participants did not like either visual message, suggesting that they would have likely chosen the opt out if it was offered. In choice sets where there was no clear preference for the participant, an opt out would improve the validity of the results and better reflect their preferences. Therefore, future DCEs exploring patient preferences for visual messaging should use a no-choice alternative.

Another limitation of this study was that some attributes might have been missed in the DCE. Attribute selection is an important step in running a DCE [31]. However, as DCEs work best when focusing on only a few attributes, other attributes may have been excluded from the survey. Therefore, there may have been attributes that influenced patient preferences but were not tested in the DCE. To ensure that all attributes are captured in the survey, it is useful to use qualitative methods, such as focus groups and interviews [33]. Owing to COVID-19, these face-to-face qualitative methods were not possible, limiting the scope of possible attributes that this DCE could have examined. In the future, given the uncertainties of the current health situation, we would like to conduct qualitative interviews over the phone or via videoconferencing to obtain valuable participant input in the development of visual messages.

In relation to the design of the visual messages, although they were developed using a systematic and iterative process, we did not pilot test them before seeking consumer feedback. Therefore, we have identified this as a limitation and, in the future, would like to test different variations of font, sizing, and layout to determine optimal combinations. In relation to the 87 participants who were sent the survey link but did not open it, there is no way of knowing their preferences, so we cannot assume that they disliked the messages from the fact that they did not open the survey.

The fourth limitation was the number of choice sets. The DCE guidelines state that the ideal number of choice tasks is 8-16 [29]. This survey was constructed with 16 choice tasks, which were thought to be an acceptable length for respondents. However, there were a number of partially completed surveys, suggesting that there were too many choice tasks. Therefore, a shorter survey may have resulted in more completions. Instead of using a full fractional design, future DCEs should use a fractional factorial design, which will allow the number of choice tasks to be reduced so that the survey is less burdensome to participants.

The strength of the investigation was limited by the small sample size. Therefore, the results are not generalizable to a wider population of people undergoing both cardiac and pulmonary rehabilitation. The small sample size also meant that data analysis was limited. In this study, a conditional logit model was used to examine the associations between the choice outcome and the visual message attributes [42]. With a larger sample size, a different model could have been used, namely, a multinomial logit model, which examines associations between the choice outcome and demographic characteristics as well as the visual message attributes. Other DCEs have used a multinomial model to examine whether preferences are influenced by age, sex, ethnicity, income, and education [28]. Future DCEs with sufficiently large sample sizes should investigate the effect of demographics on preferences. Finally, a limitation arises from the fact that this research was an additional substudy with recruitment from the ITM trial, which was not specifically designed to test visual messages [17].

### Conclusions

This study explored the preferences for lifestyle-focused visual messages in patients with CVD and chronic respiratory disease and found that message framing and image type were important characteristics. Framing was the most important factor that influenced preferences. A strong preference for gain-framed messages with real images was shown. As such, when designing visual messaging interventions for patients with chronic conditions, these attributes may influence the acceptability of messages. Owing to COVID-19 restrictions, the DCE was delivered on the web. Further research is needed to deliver visual media content using digital technology and determine whether the identified preferences actually hold. Future studies can examine the implementation of these visual messages and their effects on chronic disease rehabilitation health and behavioral outcomes.

## Abbreviations

CVD: cardiovascular disease
DCE: discrete choice experiment
ISPOR: International Society for Pharmacoeconomics and Outcomes Research
ITM: integrated text messaging
mHealth: mobile health
NCD: noncommunicable disease
OR: odds ratio

## References

[1] GBD 2017 Causes of Death Collaborators (2018). Global, regional, and national age-sex-specific mortality for 282 causes of death in 195 countries and territories, 1980-2017: a systematic analysis for the Global Burden of Disease Study 2017. Lancet.

[2] Vandenberghe D, Albrecht J (2020). The financial burden of non-communicable diseases in the European Union: a systematic review. Eur J Public Health.

[3] Carter HE, Schofield D, Shrestha R (2019). Productivity costs of cardiovascular disease mortality across disease types and socioeconomic groups. Open Heart.

[4] Patel JG, Coutinho AD, Lunacsek OE, Dalal AA (2018). COPD affects worker productivity and health care costs. Int J Chron Obstruct Pulmon Dis.

[5] (2021). Noncommunicable diseases. World Health Organization.

[6] Dalal HM, Doherty P, Taylor RS (2015). Cardiac rehabilitation. BMJ.

[7] McCarthy B, Casey D, Devane D, Murphy K, Murphy E, Lacasse Y (2015). Pulmonary rehabilitation for chronic obstructive pulmonary disease. Cochrane Database Syst Rev.

[8] Anderson L, Taylor RS (2014). Cardiac rehabilitation for people with heart disease: an overview of Cochrane systematic reviews. Cochrane Database Syst Rev.

[9] Clark RA, Conway A, Poulsen V, Keech W, Tirimacco R, Tideman P (2015). Alternative models of cardiac rehabilitation: a systematic review. Eur J Prev Cardiol.

[10] Bjarnason-Wehrens B, McGee H, Zwisler A, Piepoli MF, Benzer W, Schmid J, Dendale P, Pogosova NV, Zdrenghea D, Niebauer J, Mendes M, Cardiac Rehabilitation Section European Association of Cardiovascular PreventionRehabilitation (2010). Cardiac rehabilitation in Europe: results from the European Cardiac Rehabilitation Inventory Survey. Eur J Cardiovasc Prev Rehabil.

[11] Keating A, Lee A, Holland AE (2011). What prevents people with chronic obstructive pulmonary disease from attending pulmonary rehabilitation? A systematic review. Chron Respir Dis.

[12] Rose M, Timmons SM, Amerson R, Reimels E, Pruitt RH (2011). Facilitators and barriers in cardiac rehabilitation participation: an integrative review. J Nurse Pract.

[13] Number of mobile phone users worldwide from 2015 to 2020 (in billions). Statista.

[14] Scott-Sheldon LA, Lantini R, Jennings EG, Thind H, Rosen RK, Salmoirago-Blotcher E, Bock BC (2016). Text messaging-based interventions for smoking cessation: a systematic review and meta-analysis. JMIR Mhealth Uhealth.

[15] Siopis G, Chey T, Allman-Farinelli M (2015). A systematic review and meta-analysis of interventions for weight management using text messaging. J Hum Nutr Diet.

[16] Stephens J, Allen J (2013). Mobile phone interventions to increase physical activity and reduce weight: a systematic review. J Cardiovasc Nurs.

[17] Redfern J, Hyun K, Singleton A, Hafiz N, Raeside R, Spencer L, Carr B, Caterson I, Cullen J, Ferry C, Santo K, Hayes A, Leung RW, Raadsma S, Swinbourne J, Cho JG, King M, Roberts M, Kok C, Jenkins C, Chow C (2019). ITM support for patients with chronic respiratory and cardiovascular diseases: a protocol for a randomised controlled trial. BMJ Open.

[18] Chow CK, Redfern J, Hillis GS, Thakkar J, Santo K, Hackett ML, Jan S, Graves N, de Keizer L, Barry T, Bompoint S, Stepien S, Whittaker R, Rodgers A, Thiagalingam A (2015). Effect of lifestyle-focused text messaging on risk factor modification in patients with coronary heart disease: a randomized clinical trial. JAMA.

[19] Houts PS, Doak CC, Doak LG, Loscalzo MJ (2006). The role of pictures in improving health communication: a review of research on attention, comprehension, recall, and adherence. Patient Educ Couns.

[20] Melholt C, Joensson K, Spindler H, Hansen J, Andreasen JJ, Nielsen G, Noergaard A, Tracey A, Thorup C, Kringelholt R, Dinesen BI (2018). Cardiac patients' experiences with a telerehabilitation web portal: implications for eHealth literacy. Patient Educ Couns.

[21] Molan N, Emmanuel S, Langley T, Holloway CJ (2019). Evaluating the effectiveness of an online cardiac rehabilitation resource (www.svhhearthealth.com.au) in improving knowledge and confidence for patients with newly diagnosed cardiac conditions: a pre-experimental pilot study. Heart Lung Circ.

[22] Varnfield M, Karunanithi M, Lee C, Honeyman E, Arnold D, Ding H, Smith C, Walters DL (2014). Smartphone-based home care model improved use of cardiac rehabilitation in postmyocardial infarction patients: results from a randomised controlled trial. Heart.

[23] Forman DE, LaFond K, Panch T, Allsup K, Manning K, Sattelmair J (2014). Utility and efficacy of a smartphone application to enhance the learning and behavior goals of traditional cardiac rehabilitation: a feasibility study. J Cardiopulm Rehabil Prev.

[24] Farmer A, Williams V, Velardo C, Shah SA, Yu L, Rutter H, Jones L, Williams N, Heneghan C, Price J, Hardinge M, Tarassenko L (2017). Self-management support using a digital health system compared with usual care for chronic obstructive pulmonary disease: randomized controlled trial. J Med Internet Res.

[25] Niu Z, Jeong DC, Brickman J, Nam Y, Liu S, Stapleton JL (2020). A picture worth a thousand texts? Investigating the influences of visual appeals in a text message-based health intervention. J Health Commun.

[26] Orji R, Moffatt K (2018). Persuasive technology for health and wellness: state-of-the-art and emerging trends. Health Informatics J.

[27] Redfern J, Santo K, Coorey G, Thakkar J, Hackett M, Thiagalingam A, Chow CK (2016). Factors influencing engagement, perceived usefulness and behavioral mechanisms associated with a text message support program. PLoS One.

[28] Ramirez M, Wu S, Beale E (2016). Designing a text messaging intervention to improve physical activity behavior among low-income Latino patients with diabetes: a discrete-choice experiment, Los Angeles, 2014-2015. Prev Chronic Dis.

[29] Ryan M, Gerard K, Amaya-Amaya M (2008). Discrete choice experiments in a nutshell. Using Discrete Choice Experiments to Value Health and Health Care.

[30] Lancaster KJ (1966). A new approach to consumer theory. J Polit Econ.

[31] Bridges JF, Hauber AB, Marshall D, Lloyd A, Prosser LA, Regier DA, Johnson FR, Mauskopf J (2011). Conjoint analysis applications in health--a checklist: a report of the ISPOR Good Research Practices for Conjoint Analysis Task Force. Value Health.

[32] Coast J, Horrocks S (2007). Developing attributes and levels for discrete choice experiments using qualitative methods. J Health Serv Res Policy.

[33] Coast J, Al-Janabi H, Sutton EJ, Horrocks SA, Vosper AJ, Swancutt DR, Flynn TN (2012). Using qualitative methods for attribute development for discrete choice experiments: issues and recommendations. Health Econ.

[34] Latimer AE, Brawley LR, Bassett RL (2010). A systematic review of three approaches for constructing physical activity messages: what messages work and what improvements are needed?. Int J Behav Nutr Phys Act.

[35] Zhao X, Villagran MM, Kreps GL, McHorney C (2012). Gain versus loss framing in adherence-promoting communication targeting patients with chronic diseases: the moderating effect of individual time perspective. Health Commun.

[36] O'Keefe DJ, Jensen JD (2007). The relative persuasiveness of gain-framed and loss-framed messages for encouraging disease prevention behaviors: a meta-analytic review. J Health Commun.

[37] Thrasher JF, Anshari D, Lambert-Jessup V, Islam F, Mead E, Popova L, Salloum R, Moodie C, Louviere J, Lindblom EN (2018). Assessing smoking cessation messages with a discrete choice experiment. Tob Regul Sci.

[38] Traets F, Sanchez DG, Vandebroek M (2020). Generating optimal designs for discrete choice experiments in R: the idefix package. J Stat Softw.

[39] Redfern J, Thiagalingam A, Jan S, Whittaker R, Hackett ML, Mooney J, De Keizer L, Hillis GS, Chow CK (2014). Development of a set of mobile phone text messages designed for prevention of recurrent cardiovascular events. Eur J Prev Cardiol.

[40] Michie S, van Stralen MM, West R (2011). The behaviour change wheel: a new method for characterising and designing behaviour change interventions. Implement Sci.

[41] Hauber AB, González JM, Groothuis-Oudshoorn CG, Prior T, Marshall DA, Cunningham C, IJzerman MJ, Bridges JF (2016). Statistical methods for the analysis of discrete choice experiments: a report of the ISPOR conjoint analysis good research practices task force. Value Health.

[42] McFadden D (1974). Conditional logit analysis of qualitative choice behaviour. Frontiers in Econometrics.

[43] Manski CF (1977). The structure of random utility models. Theor Decis.

[44] Nowell LS, Norris JM, White DE, Moules NJ (2017). Thematic analysis: striving to meet the trustworthiness criteria. Int J Qual Methods.

[45] Rothman AJ, Salovey P (1997). Shaping perceptions to motivate healthy behavior: the role of message framing. Psychol Bull.

[46] Gallagher KM, Updegraff JA (2012). Health message framing effects on attitudes, intentions, and behavior: a meta-analytic review. Ann Behav Med.

[47] Tversky A, Kahneman D (1981). The framing of decisions and the psychology of choice. Science.

[48] Covey J (2014). The role of dispositional factors in moderating message framing effects. Health Psychol.

[49] Hurlbert A, Ling Y (2017). Understanding colour perception and preference. Colour Design (Second Edition).

[50] Joffe H (2008). The power of visual material: persuasion, emotion and identification. Diogenes.

[51] Richards AS, Hample D (2015). Facial similarity mitigates the persuasive effects of source bias: an evolutionary explanation for kinship and susceptibility to influence. Commun Monogr.

[52] Buller MK, Bettinghaus EP, Fluharty L, Andersen PA, Slater MD, Henry KL, Liu X, Fullmer S, Buller DB (2019). Improving health communication with photographic images that increase identification in three minority populations. Health Educ Res.

[53] Neubeck L, Lowres N, Benjamin EJ, Freedman SB, Coorey G, Redfern J (2015). The mobile revolution--using smartphone apps to prevent cardiovascular disease. Nat Rev Cardiol.

[54] Clark M, Kelly T, Deighan C (2011). A systematic review of the Heart Manual literature. Eur J Cardiovasc Nurs.

[55] DeWalt DA, Malone RM, Bryant ME, Kosnar MC, Corr KE, Rothman RL, Sueta CA, Pignone MP (2006). A heart failure self-management program for patients of all literacy levels: a randomized, controlled trial [ISRCTN11535170]. BMC Health Serv Res.

[56] Bucknall CE, Miller G, Lloyd SM, Cleland J, McCluskey S, Cotton M, Stevenson RD, Cotton P, McConnachie A (2012). Glasgow supported self-management trial (GSuST) for patients with moderate to severe COPD: randomised controlled trial. BMJ.

[57] Sartori AC, Rodrigues Lucena TF, Lopes CT, Picinin Bernuci M, Yamaguchi MU (2020). Educational intervention using on medication adherence in hypertension and diabetes patients: a randomized clinical trial. Telemed J E Health.

[58] Mühlbacher AC, Kaczynski A, Zweifel P, Johnson FR (2016). Experimental measurement of preferences in health and healthcare using best-worst scaling: an overview. Health Econ Rev.

